# Space allowance: a tool for improving behavior, milk and meat production, and reproduction performance of buffalo in different housing systems—a review

**DOI:** 10.1007/s11250-022-03247-y

**Published:** 2022-08-15

**Authors:** Mohamed I. El Sabry, Obaida Almasri

**Affiliations:** 1 Department of Animal Production, Faculty of Agriculture, Cairo University, El-Gamma street, Giza, 12613 Egypt; 2 General Commission for Scientific Agricultural Research, Damascus, Syria

**Keywords:** Buffalo population, Stocking density, Pregnancy rate, Milk, Daily gain, Social behavior

## Abstract

Buffalo population has dramatically increased during the last two decades, especially in tropical and subtropical regions. Although buffalo are important milk and meat-producing animal, still practices of buffalo farming and welfare aspects are not well established. Housing system and stocking density are significant factors that affect the welfare and production of animals; however, no space allowance standards have been demonstrated for buffalo at different ages. This review article presents the following: (1) an overview of buffalo subtypes and the geographical distribution of buffalo populations and their production; (2) the effect of housing systems and space allowance on the social behavior and welfare indices; (3) the effects of space allowance on milk production and growth performance of buffalo; and (4) the relationship between space allowance and reproductive performance. Although the limited data in this area of research, it can be driven that a larger space allowance with access to a pool, especially during the hot season, maintains buffalo production at optimal levels. Moreover, optimal floor space improves the welfare and social indices of buffalo; however, there are discrepancies in aggressive and agonistic behavior results. Surprisingly, the reproductive performance of buffalo was not affected by space allowance. Therefore, further research is needed to identify the impact of the housing aspects, including space allowance and enrichment tools, on the productive performance, and welfare indices of buffalo. This would assist in implementing welfare-economic standards for buffalo production and reveal the potentiality of this eco-friendly animal.

## Introduction

*Bubalus bubalis* is the scientific name of the domesticated water buffalo (Abd El-Salam and ElShibiny 2011), which is classified into two subtypes (Yue et al. 2013). The water and swamp types have different chromosome karyotypes, and morphological and behavioral characteristics, e.g., water buffalo have 50 chromosomes, while swap type has only 48 (Yilmaz et al. 2012) as well as the body weight of water buffalo is heavier (450 to 1000 kg/head) than that of swamp ones (325 to 450 kg). It is worthy to mention that water buffalo present 79.5% and swamp buffalo present 20.5% of the global buffalo population in the world (Perera 2008).

Buffalo are adapted to live in the hot environment, because of their morphological features such as melanin-pigmented skin and low hair density. These morphological characteristics protect buffalo against ultraviolet rays and help in getting rid of heat stress by convection and radiation (Marai et al. 2009). Recently, molecular studies indicated that the higher heat tolerance of buffalo breeds may be due to their historical origin in a hot environment (Mokhber et al. 2019). Moreover, buffalo can be productive under harsh conditions because of their ability to convert poor-quality high fiber feedstuffs into high-quality products and their high resistance to diseases (Guerrero-Legarreta et al. 2020). Also buffalo have a longer lifespan (about 30 years) and productive life (ranges 18–25 years) in comparison with those of beef cows (12 years, 7–10 years, respectively) and dairy cows (4.5–6 years, 3–4 years, respectively) (Naveena and Kiran 2014; Ramsay et al. 2017; De Vries and Marco 2020).

From social and behavioral perspectives, buffalo are calm, docile, intelligent, curious, and easy-to-adapt animals (Wanapat and Kang 2013). Also, buffalo are considered an eco-friendly animal compared to other ruminants due to their lower methane production, e.g., buffalo produce 157 g methane/daily/head, which is 58% lesser than that of cows (376 g/daily/head) (Sarubbi et al. 2013; Appuhamy et al. 2016). Therefore, big attention has been paid to enhancing buffalo production, especially with the increase in temperatures and shortage in water resources due to global warming.

Stocking density (SD) rate is an important animal management and welfare aspect that helps animals to overcome the negative impacts of climate changes. In addition, it enables animals to express their potential productive characteristics. However, the available studies on the effects of SD rate/space allowance on buffalo were few and not always conclusive due to the interaction of other experimental factors such as the genetic background of the breed (Abdel-Rahman et al. 2008) and different housing systems.

Therefore, the purpose of this review is to present the following: (1) an overview of the geographical distribution of buffaloes’ populations and their milk and meat production; (2) the effects of housing systems and SD rates on the social behavior and welfare indices; (3) the effects of SD on milk production and growth performance parameters of buffalo; and (4) the relation between SD rate and reproductive performance.

## Distribution of buffalo populations

As a result of buffalo breeding programs, the statistics showed that the annual increase in the global population of buffalo during the last two decades is about 2% (Zicarelli 2020). Also, Minervino et al. (2020) showed that buffalo are not only spread in hot regions, but also small buffalo populations exist in EU countries.

**Fig. 1:**
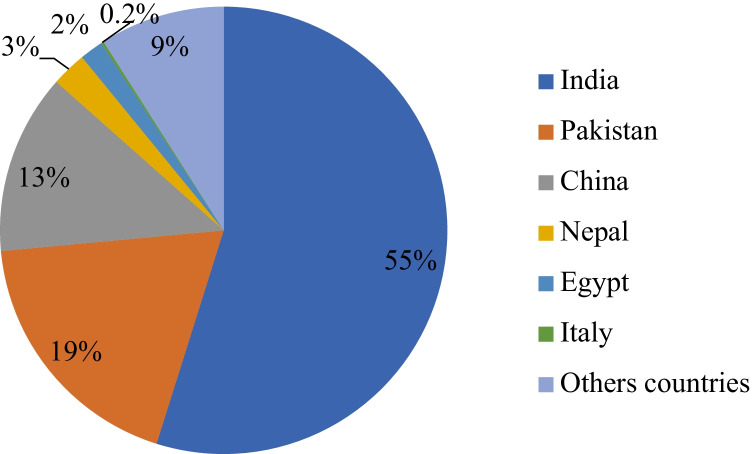
The distribution of buffalo populations worldwide (Minervino et al., 2020)

From a productive perspective, the water buffalo is a valuable multipurpose animal (Abd El-Salam and El-Shibiny 2011; De la Cruz-Cruz et al., 2014) since its meat, milk, horns, and skin can be utilized. In addition, in many parts of Asia, the domesticated water buffalo is often called “the living tractor of the East” since buffalo are used in field draft and transportation (Chantalakhana and Bunyavejchewin 1994; Bakkannavar et al. 2010). Therefore, it is normal to find the largest buffalo population in Asian countries, e.g., India, Pakistan, and China (Fig. 1).

## Stocking density affects social behavior and welfare indices

The SD rate is the number of animals that are growing in a specific area. Since the welfare-cost balance has become a significant aspect of animal production, the space requirement for animals becomes not only a matter of mass production but also a matter of health and welfare issues. Therefore, it is important to present the effects of SD rate/space allowance on buffaloes’ behavior and welfare status.

Buffalo are one of the social animals that prefer to live in a herd. However, heat waves, diseases, and restricted space stresses can lead to oxidative stress (Odore et al. 2011; El Sabry et al.^2021^; ^2022^), which is commonly associated with the incidence of several health problems, such as retained placenta, udder edema, and mastitis. Consequently, this deterioration in health status can lessen the production performance of animals (StarvaggiCucuzza et al., 2014).

From a welfare point of view, animals under optimal environmental conditions can easily express their appropriate species-specific behaviors. While under inadequate conditions, e.g. under restricted space, animal welfare status will be negatively affected. For example, animals could change their habitats, and their physical and psychological characteristics such as reducing their movement activities (Maton and Daelemans 1989; Hanlon et al. 1994) and appearing signs of stress (Fisher et al. 1997; Grasso et al. 1999).

Thus, for improving the well-being status of animals, Vaarst et al. (2001) suggested that adding enrichment tools and increasing space allowance make animals’ lives better. In context, Grasso et al. (1999) reported that the 7- to 10-day-old weaning calves raised in larger pens of 2.6 m^2^ indoor + 2 outdoor m^2^ spent less time at rest and slept with more legs extended compared to those of calves raised in 2.6 m^2^ and 1.5 m^2^ (*P* ≤ 0.01 and *P* ≤ 0.001).

Also, Napolitano et al. (2004) and Abdel-Rahman et al. (2008) showed the effect of floor space in relation to buffalo's body surface area on a range of behavioral parameters. They calculated the space allowed/head as a percentage of the animal’s body surface area in square meter (Body surface area (m^2^) = 0.12 body weight (kg)^0.60^ according to Hurnik and Lewis (1991). The allowed floor space/head for calves in the 1st group and 2nd groups were 50 (1.1 m^2^) and 90% (2 m^2^) of the body surface area (m^2^). They found that the calves of the 1^st^ group lay with a lower number of outstretched legs and ruminating activities compared to those of calves in the 2^nd^ group. Calves of the 1st group showed higher agonistic interactions and standing more frequently compared to those of calves in the 2^nd^ group (*P* < 0.001). Moreover, Napolitano et al. (2004) found that the proportions of calves’ idling (*P* < 0.01) and lying idle (*P* < 0.001) were higher in the 2^nd^ group than those in the 1^st^ one. Moreover, the 1^st^ group of animals displayed longer movement time, a greater number of galloping events, and more vocalization. Furthermore, Abdel-Rahman et al. (2008) indicated that the blood cortisol and glucose levels of calves in the 1^st^ group were significantly higher (*P* < 0.01) than those in the serum of calves of the 2^nd^ ones. This increase in blood cortisol may be associated with higher ACTH hormone secretion.

The effect of floor space on buffalo heifers was studied by Grasso et al. (2003). They evaluate the effect of two space allowances (2.3 indoor slatted floor and 2.3 indoor slatted floor + 15 outdoor yards m^2^/head) on social behavior and humoral immunity of heifers (19 months old and weight 390 kg). The authors found that the heifers with an outdoor paddock lay with a higher number of outstretched legs significantly (*P* < 0.01) than those provided with less free space, whereas De Rosa et al. (2007) indicated that the buffalo heifers that reared under an inxtensive system (housed in an indoor slatted floor pen 3 m^2^/head) with an outdoor paddock (3 m^2^/head) based on pasture seem to be a valid method to promote welfare and sustainability of buffalo heifer. These results agree with the findings of Napolitano et al. (2004) and Abdel-Rahman et al. (2008),who referred to the high sensitivity of young animals to environmental stressors.

In milking buffalo, De Rosa et al. (2009) raised two groups of buffalo under a free stall open-sided barn with a concrete floor. The space allowance was 10 m^2^/head in the 1^st^ group, while in the 2^nd^ group buffalo had 10 m^2^/head with access to an outdoor yard (36 m^2^/head) and a pool. They found that a smaller proportion of buffalo from the 2^nd^ group (14%) was observed lying compared to the proportion of lying buffalo from the 1^st^ group (55%; *P* < 0.001). This result could be due to the buffalo from the 2^nd^ group resting while wallowing (48%; i.e.  lying in the pool). There was a significant positive correlation (*r* = 0.41, *P* < 0.05) between temperatures degree and the proportion of buffalo in the pool. They also found that a greater space allowance with a pool had enhanced the social behavior of buffalo in the 2^nd^ group, increasing social licking (15 *Vs*. 9%), social interactions (sniffing and nuzzling) (12 vs. 7% and 15 *Vs*. 9%, respectively), and investigative activities (10 vs. 4%), but reducing idling (44 *Vs.*51%).

Under an intensive system, buffalo show high-stress signs, high agonistic behaviors, and low time for walking (Cavallina et al. 2008), which are due to smaller space allowance. In context, Tripaldi et al. (2004) studied the effect of the housing system on some behavioral and physiological traits. The 1^st^ group of buffalo were housed in a loose open-sided barn with a concrete floor and 10 m^2^ per head, while the 2nd one was housed in a similar barn but they had an access to the pool and an outdoor yard with 500 m^2^ per head. They found that the proportion of cortisol levels, idling, and standing at the fodder were greater in the 1^st^ group than in the 2nd one (*P* < 0.001). On the other hand, a higher percentage of buffalo in the 2^nd^ group was standing in the sunny area compared to that in the 1^st^ group (*P* < 0.001). This result indicated that the intensive system adversely impacts welfare status of buffalo.

Likewise, feeding behavior was affected by the production system; a higher proportion of 1^st^ group buffalo were found eating early in the day when the environmental temperature was lower than the rest of the day. In the 2^nd^ group, at the same period of the day (07:30 to 09:30 h) more buffalo grazed in the yard; these observations are consistent with the well-known phenomenon that high temperatures depress ingestive activities. However, the existence of pools facilitated thermoregulation of the buffaloes in the second group; they were found in the sun in a higher proportion than were 1^st^ group buffalo. Similarly, Grasso et al. (1999) reported that the buffalo raised under a free-range system spent higher time walking (*P* ≤ 0.05), feeding (*P* ≤ 0.01), and standing (*P* ≤ 0.01), and reduced their agonistic behavior (*P* ≤ 0.05) compared to ones that were raised in limited spaces. The authors suggested that these alterations in the behavior of buffaloattributed to changes in some physiological responses as a respond to the space restriction stress.

In terms of animal-human contact, Tripaldi et al. (2004) reported that the lactating buffalo raised under an extensive system; they showed less self-maintenance and grooming activities, low excitability and anxious temperament, which made the human- buffalo contact difficult. During the production stage, there may be problems in terms of welfare, since feeding exclusively on grasses does not meet the animals’ energy requirements.

Finally, the effects of housing system on the welfare of some species-specific natural behavior are summarized in Table (1).

**Table 1:** The effect of indoor housing systems and stocking density on species-specific natural behaviors and welfare indices of lactating buffalo

Studied parameters	Intensive system	Semi-intensive system	Extensive system	References
Allowed space	Barn with a concrete floor and 10 m^2^/head	Barn with a concrete floor and 10 m^2^/head + outdoor yard (36 m^2^/head) + free access to pool	Open-sided barn + outdoor yard 500 m^2^/head + free access to pool	
Standing	No difference	No difference	–	De Rosa et al. (2009)
Time for walking	↓	–	–	(Cavallina et al. 2008)
Idling	↑	↓	↓	Tripaldi et al. (2004), De Rosa et al. (2009)
Grooming activities	↓	–	↑	Tripaldi et al. (2004)
Investigative activities	↓	↑	–	De Rosa et al. (2009)
Restless during handling	↑	–	↓	Tripaldi et al. (2004)
Licking social	↓	↑	–	De Rosa et al. (2009)
Sniffing and nuzzling	↓	↑	–	De Rosa et al. (2009)
Aggression social	↓	↑		De Rosa et al. (2009)
Scores for cleanliness	↓	–	↑	De la Cruz-Cruz et al. (2014)
Grazing and bathing activities	–	–	↑	Tripaldi et al. (2004), Napolitano et al. (2013)
Wallowing	–	–	↑	Tripaldi et al. (2004)
Location in the sun	↓	–	↑	Tripaldi et al. (2004)

↑ = increase, ↓ = decrease

Age of lactating buffalo: ≥ 2 years. Live body weight: ranged from 610 to 667.5 kg

Conclusively, allowing larger floor space for milking buffalo would help them to express their species-specific natural behaviors and may avoid crowding stress influences. Moreover, it is necessary to enrich the knowledge about the effects of SD on the buffalos' behavior during the fattening period, which will assist in determining the optimal allowed space for increasing the meat yield of buffalo

## Effects of stocking density rate on productive traits

**Fig. 2:**
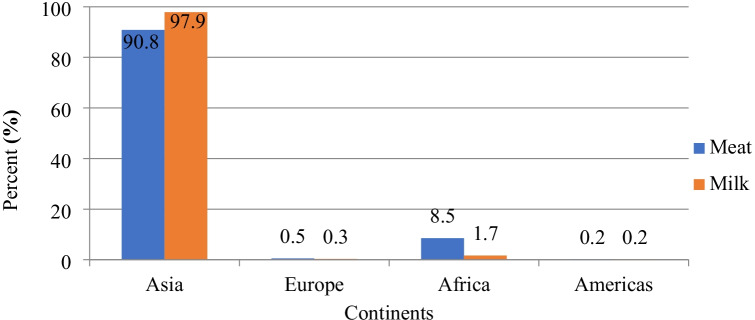
Distribution of buffalo milk and meat production in the world (Minervino et al. 2020; Di Stasio and Brugiapaglia 2021)

**Fig. 3:**
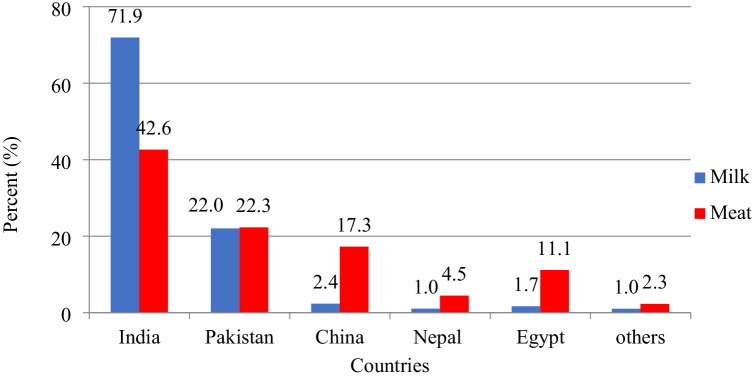
The top buffalo milk and meat-producing countries (Naveena and Kiran 2014; Minervino et al. 2020)

Improving environmental conditions, including SD/space allowance, results in the improvement of production traits for animals (Tripaldi et al. 2004; Keane et al. 2017; Ha et al. 2018; Sharpe and Kenny 2019; Park et al. 2020; Xiao et al. 2020; El Sabry et al. 2022). Recently, there is much interest in buffalo production due to its valuable products and harsh environment adaption (Addeo et al. 2007). Minervino et al. (2020) indicated that buffalo populations in Asian countries represent around 97.9% of milk buffalo and 90.8% of meat buffalo in the world (Minervino et al. 2020; Di Stasio and Brugiapaglia 2021). India occupies the 1^st^ position in milk and meat production in the world. India’s contribution to the world’s buffalo milk and meat production is about 71.9 and 42.6%, respectively (Naveena and Kiran 2014; Minervino et al. 2020), whereas Pakistan occupies the 2^nd^ position in buffalo milk and meat-producing countries with about 22 and 22.3% of the global buffalo milk and meat production, respectively (Figs. 2 and 3).

## Milk production

The water buffalo occupy fundamental species in the world in terms of milk yield, after dairy cattle (Coroian et al. 2013). But, the value of buffalo milk is the highest compared to other lactating animals. Thus, buffalo milk is used in high-quality cheese production, e.g. mozzarella (Aspilcueta-Borquis et al. 2012; Senosy and Hussein 2013). Furthermore, buffalo milk has a high nutritional value because it contains low cholesterol, sodium, and potassium, but high concentrations of calcium, phosphorous, and A and E vitamins. Also, it has higher dry matter and total solids compared to cows’ milk. This characterization increases the preference for buffalo’s milk compared to cow’s milk (Deng et al. 2019; Mota-Rojas et al. 2020; Guerrero-Legarreta et al. 2020).

In general, the type of housing system affects the productive performance of buffalo (De Rosa et al. 2009). In addition, there is a positive correlation between allowed floor space and milk yield (Zicarelli et al. 2005). They found that the space allowance of 22 m^2^/head improved milk production. Similarly, in an intensive system, Vecchio et al. (2009) indicated that the milk yield of lactating buffalo raised in paddocks with an allowed floor space of 20 m^2^/head was significantly higher than that of buffalo with an allowed floor space of 15 m^2^/head. Authors suggested that greater allowed space (20 m^2^/head) leads to a reduction of fooder access competition, which probably reduced fat mobilization and positively affected milk synthesis.

De Rosa et al. (2009) reported that buffalo raised in a free stall open-sided barn with a concrete floor where they received 10 m^2^/head as space allowance produced less milk (10.8 kg/day) than that of buffalo reared in an outdoor yard (36 m^2^/head) and a concrete pool of 208 m^2^ (11.7 kg/day). However, the milk protein and fat were not affected by the housing system or allowed floor space. They also showed the obvious effect of the pool, especially at high temperatures. The pool helps the buffalo to regulate their body temperature. The authors recommended that the presence of a pool and allowed floor space would improve both welfare and milk production.

Under the open-air system, De la Cruz-Cruz et al. (2014) mentioned that the milk production of water buffalo was higher by 0.35 kg compared to buffalo under limited space. They suggested that the feeding time of grazing animals was more than that of buffalo in the stables. Also, Salzano et al. (2019) reported that the lactating buffalo were reared in the 1^st^ group (10 m^2^/head) and produced less milk compared to those in the 2^nd^ group (15 m^2^/head). But, the percentage of milk fat, protein, and lactose in both groups were similar.

Conversely, Salzano et al. (2017) found the daily milk yield was not affected by SD/space allowance, e.g. the milk yield of buffalo kept at 22 m^2^/head or 10 m^2^/head was similar. This result agreed with findings by Vecchio et al. (2012) and Balestrieri et al. (2013). Finally, it can be suggested that the discrepancies in the previous results are due to the different production systems, diets, ambient temperature……*etc*. So, considering these variables in future investigation will assist in finding out the optimal environment for milking buffalo.

## Meat production

Asian buffalo (*Bubalus bubalis*) has the potential for high yield meat production (Naveena and Kiran 2014), especially in tropical and subtropical environments, where the high temperature and pastures are poor in terms of their quality (Ranjan, 1992). Additionally, the biological value of buffalo meat is high because it contains greater protein and iron, and lower intramuscular fat, caloric, cholesterol, and triglyceride contents compared to beef meat. Moreover, buffalo meat has high-quality characteristics, *e.g.* dark red color, good marbling, low connective tissue content, desirable texture, and high protein content (Kandeepan et al. 2013). Finally, there are no religious restrictions on buffalo meat consumption compared to some other animals (Cruz-Monterrosa et al. 2020).

The type of housing system may affect the meat production of buffalo. For example, Spanghero et al. (2004) reported that the average daily gain of male buffalo, in stables, declined during the fattening period. Conversely.

 Grasso et al. (2003) found that the average daily weight gain was similar for heifers (19 months old) that were raised under outdoor paddock (2.3 indoor + 15 outdoor m^2^/head) and heifers that were raised under limited space (2.3 indoor m^2^/head). Under experimental conditions, the authors concluded that the buffalo heifers were not affected by space allowance.Regarding the allowed space, Napolitano et al. (2004) indicated that the daily weight gain was not affected by the space allowance, when two groups of weaned buffalo were raised in 1.1 m^2^/head for the 1st group or 1.9 m^2^/head for the 2^nd^ group during the first month and received 1.9m^2^/ head for the 1^st^ group or 3.4 m^2^/head for the 2^nd^ group during last month of the experiment. However, we suggest that the available literature in this area is not enough to determine the optimal allowed floor space for meat type buffalo.

## Effects of stocking density on reproductive traits

The stress, which results from reduced increasing SD, can adversely affect reproduction parameters of different species such as buffalo (Di Palo et al. 2001), sheep (Holmøy et al. 2012; Bøe and Jørgensen, 2012), sow (Hemsworth et al. 2013), cattle (Miranda-de la Lama et al. 2013), and quail (El Sabry et al. 2022).

In buffalo, De Rosa et al. (2009) found that the reproductive interactions (bull or female sniffing the genital region or mounting) of buffalo that were reared in the 1^st^ group (a free stall open-sided barn with an outdoor yard system (36 m^2^/head) and a concrete pool of 208 m^2^) were lower than that of ones reared in the 2^nd^ group limited space (a free stall open-sided barn with a concrete floor with space (10 m^2^/head)). Reproductive interactions were 0.09 vs. 0.11 in the 1^st^ and 2^nd^ groups, but self-grooming was higher in the 1^st^ group than in the 2^nd^ one (0.84 vs. 0.68, respectively). On the other hand, the pregnancy rates and the number of days open in both groups were similar. The pregnancy rates were 68.0 and 68.7, respectively and the number of days open was 91.8 and 91 days, in the 1^st^ and 2^nd^ groups, respectively. Moreover, it is noteworthy that the average ambient temperatures may not be high enough to affect the reproductive performance of buffalo in Italy.

Similarly, Salzano et al. (2017) indicated that the allowed space had no significant effect on the reproductive performance of Italian buffalo. They reported that buffalo in the 1st group (high density) were reared in open yards that allowed 10 m^2^/head, while those in the 2nd group (low density) were reared in 22 m^2^/head and showed similar conception rate, late embryonic mortality, and fetal mortality.

Also, Salzano et al. (2019) reported that the pregnancy rate and days open of lactating buffalo were not affect by different space allowances (10 and 15 m^2^/head in 1st and 2nd groups, respectively). They were 73.1% and 147 days in the 1^st^ group, compared to 69.2% and 138 days, in the 2^nd^ group, respectively. Moreover, it was noticed that the incidence of lameness increased in the restricted space group (Table 2).

**Table 2:** The effects of housing system and space allowances on the milk and meat production and reproductive traits of buffalo

Studied parameters	Intensive system	Semi-intensive system	Extensive system	References
Allowed space	Barn with a concrete floor and 10 m^2^/head	Barn with a concrete floor and 10 m^2^/head + outdoor yard (36 m^2^/head) with a free access to pool	Open-sided barn with 500 m^2^/head with a free access to pool	
Milk production	↓	↑	–	De Rosa et al. (2009), De la Cruz-Cruz et al. (2014)
Meat production	↑↓	–	–	Borghese (2013), Spanghero et al. (2004)
Pregnant rate, open days	No difference	No difference	–	De Rosa et al. (2009), Salzano et al. (2017), Salzano et al. (2019)
Immunological status	↓	–	↑	Grasso et al. (1999)
Claw conformation	–	–	↑	Loberg et al. (2004)

↑ = increase, ↓ = decrease

## Conclusion

This review identified a wide range of scientifically documented examples of the effect of SD on the welfare and health and productive indices. Important tips could be concluded from these scientific examples:

- A larger floor space increases movement and investigative activities, and social interactions. While it decreases idling, aggressive behavior, and lameness problem.
- Production system enrichment with an outdoor yard (36 m^2^) and pool improved welfare degree and social behavior indices of buffalo.
- Considering an economic-welfare balance, it can be suggested that the optimal space allowances for weaning buffalo and heifers are 2.6 m^2^ indoor + 2 outdoor m^2^ and 2.3 m^2^ indoor slatted floor + 15 outdoor yards m^2^/head, respectively.
- In semi-intensive rising systems, accessing to a pool and an ample outdoor yard according to the habits of buffalo can benefit the behavior, welfare, and milk production of lactating buffalo, especially in hot regions.
- Surprisingly, the reproductive traits were not enhanced by increasing floor space from 10 to 20 m^2^/head.
- Finally, it can be seen that buffalo are social animals that live in nature and have species specific habits. Therefore, the raising style must be adjusted according to the desired objectives, with welfare aspects in mind.

## Data Availability

Not applicable.
