# Correction to: Space allowance: a tool for improving behavior, milk and meat production, and reproduction performance of buffalo in different housing systems—a review

**DOI:** 10.1007/s11250-022-03312-6

**Published:** 2022-09-23

**Authors:** Mohamed I. El Sabry, Obaida Almasri

**Affiliations:** 1 Department of Animal Production, Faculty of Agriculture, Cairo University, El‑Gamma street, Giza, 12613 Egypt; 2 General Commission for Scientific Agricultural Research, Damascus, Syria

**Correction to: Tropical Animal Health and Production (2022) 54:266**

**https://doi.org/10.1007/s11250-022-03247-y**

In the original version of this article, the name of the first author is missing the middle initial “I.” in the HTML version of the paper. The correct name is shown above.

The original article has been corrected.

